# Lung Cancers: Parenchymal Biochemistry and Mechanics

**DOI:** 10.3390/cells13050427

**Published:** 2024-02-29

**Authors:** Yves Lecarpentier, Bruno Tremblay, Christèle Locher, Olivier Schussler, Alexandre Vallée, Christophe Locher, David Pho

**Affiliations:** 1 Centre de Recherche Clinique, Grand Hôpital de l’Est Francilien, 77100 Meaux, France; 2 Service de Chirurgie Thoracique et Vasculaire, Grand Hôpital de l’Est Francilien, 77100 Meaux, France; btremblay@ghef.fr; 3 Service de Pneumologie, Grand Hôpital de l’Est Francilien, 77100 Meaux, France; 4 Département de Chirurgie Thoracique, Hôpital Cochin, Assistance Publique-Hôpitaux de Paris, 75014 Paris, France; 5 Department of Clinical Research and Innovation, Foch Hospital, 92150 Suresnes, France; 6 Service d’Hépato-Gastro-Entérologie, Grand Hôpital de l’Est Francilien, 77100 Meaux, France; 7 Service d’Anatomie et Cytologie Pathologiques, Grand Hôpital de l’Est Francilien, 77600 Jossigny, France

**Keywords:** lung cancer, mechanics, non-muscle myosin, β-catenin, canonical WNT pathway, myofibroblast

## Abstract

Parenchyma of pulmonary cancers acquires contractile properties that resemble those of muscles but presents some particularities. These non-muscle contractile tissues could be stimulated either electrically or chemically (KCl). They present the Frank–Starling mechanism, the Hill hyperbolic tension–velocity relationship, and the tridimensional time-independent tension–velocity–length relationship. Relaxation could be obtained by the inhibition of crossbridge molecular motors or by a decrease in the intracellular calcium concentration. They differ from muscles in that their kinetics are ultraslow as evidenced by their low shortening velocity and myosin ATPase activity. Contractility is generated by non-muscle myosin type II A and II B. The activation of the β-catenin/WNT pathway is accompanied by the high level of the non-muscle myosin observed in lung cancers.

## 1. Introduction

The parenchyma of lung cancers acquires particular contractile properties. This is due to the appearance of a contractile protein, the non-muscle myosin (NM) [1]. This protein is found in cancer cells themselves and cancerous stroma. The goal of this study was to highlight their contractile properties and to determine similarities and differences with those of sarcomeric and smooth muscles. Most fundamental contractile properties described in the non-muscle lung parenchyma of cancer were observed in normal muscles. Thus, the Frank–Starling phenomenon [2,3], the hyperbolic Hill tension–velocity relationship [4], the three-dimensional time-independent tension–velocity–length relationship [5], and relaxation mechanisms were observed in non-muscles. However, two significant differences appeared, namely the very low level of tension and the ultraslow kinetics of the non-muscle myosin (NMIIA and IIB).

NMIIA and IIB appear to be the molecular motor of non-muscle tissues, and are characterized by particularly slow contractile properties when compared with those of muscles [1,6]. This is the case for the placenta [7,8] and for bio-engineered tissues such as with human-bone-marrow-derived mesenchymal stem cells seeded in a collagen scaffold and with TGH-β [9].

Moreover, in cancers, multiple targets of the canonical WNT/β-catenin pathway have been reported and an overview of checkpoints of this signaling has been described [10]. The canonical WNT/β-catenin pathway is a key element in the genesis and development of numerous human cancers. Three major cyclic processes, namely the cell division cycle [11,12,13], the immune cycle [14], and circadian rhythms (CRs) [15], become highly disturbed due to the upregulation of this signaling. In addition, the upregulated dysfunction of the canonical WNT signaling is involved in the development of the cancer stroma [16,17], the Warburg glycolysis [18], inflammation, and fibrosis [19,20]. The upregulation of the non-muscle myosin and the canonical WNT/β-catenin signaling are often associated as in cancers. Their association appears in shaping organs by Wingless-int/Notch/non-muscle myosin [21]. In non-muscle myosin II B-ablated and mutated mice, the ablation of non-muscle myosin (NM IIB) during embryonic development leads to marked enlargement of the cerebral ventricles and the destruction of brain tissue, due to hydrocephalus [22]. In lung cancer, the upregulation of β-catenin and of NMIIA and NMIIB is associated with fibrosis.

## 2. Materials and Methods

### 2.1. Ethics

This study received favorable advice from the “Comité de Protection des Personnes, Sud-Ouest et Outre-Mer”, n° IDRCB. 2018-A01134-51 (dossier 1-18-42). Written consent was signed by the patients.

### 2.2. Specimen

The tissue samples were examined by the Service d’Anatomie et Cytologie Pathologiques, Grand Hôpital de l’Est Francilien, Jossigny, France. Surgical specimens were obtained by excision from 21 patients (age, 44 to 88 years); there were 14 cases of adenocarcinoma (ADK), 5 cases of squamous cell carcinoma, 1 typical carcinoid tumor, and 1 metastasis from prostatic cancer.

### 2.3. Immuno-Histochemistry

Tissue specimens were taken immediately after surgical resection, fixed in 10% buffered formalin, and embedded in paraffin. Standard 4-μm-thick sections were stained with hematoxylin, eosin, and saffron (HES), prior to the immune-histochemical study. The expression of these markers was detected by immuno-histochemistry. 

Immuno-histochemistry was performed on Ventana Benchmark XT (Procedure XT ultraview DAB) using the primary polyclonal antibodies MYH9 (NM-IIA, ElabScience (Huston, TX, USA), dilution 1:100; catalog no. E-AB-62837) and MYH10 (NM-IIB, ElabScience (Huston, TX, USA), dilution 1:100; catalog no. E-AB-63609), the mouse monoclonal antibody β-catenin (β-catenin, Cell marque, (Rocklin, CA, USA) dilution 1:25; catalog no. 224M-14), and smooth muscle actin (SMA clone 1A4, Zytomed Systems, (Berlin, Germany) dilution 1:100). For positive controls, we used stomach specimens (MYH9, SMA) and testis specimens (MYH10 and β-catenin). 

### 2.4. Mechanical Studies

#### 2.4.1. Experimental Protocol

Pulmonary human samples were extracted and quickly transported to the laboratory of mechanics where they were mounted in a tissue chamber containing a Krebs–Henseleit solution, and bubbled with 95% O_2_–5% CO_2_ to maintain a pH at 7.4 at room temperature. Lo was the initial length of samples corresponding to basal tone. The contractile activity of the pulmonary sample was induced either after tetanic electrical stimulation or after exposure to KCl at a 0.05 M concentration. The tetanic conditions were train period: 5 s; train duration: 2 s; stimulus frequency: 100 Hz; stimulus duration: 5 ms; tetanus duration: until reaching a plateau.

**Figure 1:**
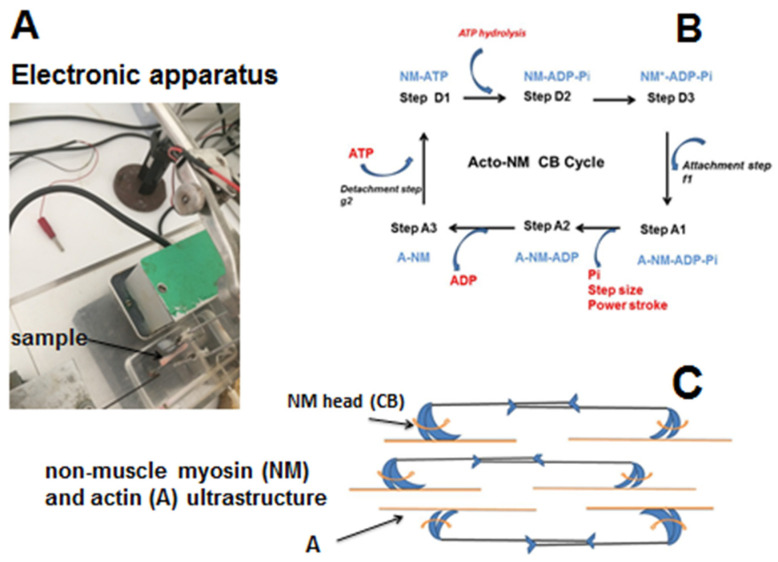
Electronic set-up, SMA-NMIIA-B cycle, and schematic SMA-NM IIA-B sliding mechanism. (**A**) Electronic set-up showing the experimental non-muscle system. (**B**) NMII-CB cycle. The CB cycle was composed of six different conformational steps, i.e., three detached steps (D1, D2, and D3) and three attached steps (A1, A2, and A3). The myosin molecular motor was non-muscle myosin type IIA (NMIIA) and IIB (NMIIB). The power stroke was characterized by the generation of a unitary CB force and the CB step. The power stroke occurred with a tilt of the NMII head and produced a force of a few pico Newtons and a displacement of a few nanometers. Importantly, the kinetics of NMII were extremely slow. (**C**) NMII binds with actin through the head domain of the heavy chain. Importantly, NMIIA and B molecules assembled into bipolar filaments, allowing myosin to slide along actin in an anti-parallel manner. A tilt of the motor domain enabled a conformational change that moved actin filaments in an anti-parallel manner. The ATP molecule formed a bond with the NMII-ATPase site located on the motor domain. This allowed the dissociation of actin from the NMII head. ATP was then hydrolyzed and subsequently NMII formed a bond with actin. Then, the power stroke occurred with a tilt of the NMII head, which generated a CB single force and a displacement of a few nanometers. ADP was then released from the actin–NMII complex. A new ATP molecule dissociated actin from the motor domain, and a new CB cycle began.

The electromagnetic lever system (Figure 1A) has been described earlier [7]. The maximum unloaded shortening velocity (Vmax, in Lo. s^−1^) and peak isometric tension of samples were measured from the (T–V) relationship by means of 6–8 afterload contractions from zero-load to isometric tension [4], fitted according to A.V. Hill’s equation:

(T + a) (V + b) = [To + a] b(1)

where -a and -b are the asymptotes of the Hill hyperbola. The G curvature of the T–V relationship was equal to To/a = Vmax/b [4].

#### 2.4.2. A. Huxley Formalism

A. Huxley’s crossbridge (CB) model remains the most commonly accepted model for calculating myosin kinetics, both in striated and smooth muscles and in non-muscle contractile systems. The Huxley equations [23] allow calculations of the molecular properties of contractile CBs. The rate of total energy release (E_Hux_) and isotonic tension (P_Hux_) as a function of muscle velocity (V) were obtained as follows:

E_Hux_ = (Ne) (h/2 l) (f_1_/(f_1_ + g_1_)) [g_1_ + f_1_ (V/Φ) [(1 − exp (−Φ/V)]] (2)

P_Hux_ = N (sw/2 l) (f_1_/(f_1_ + g_1_)) [1 − (V/Φ) [(1 − exp (−Φ/V)) (1+ (1/2) ((f_1_ + g_1_)/g_2_)^2^ (V/Φ]] (3)

where f_1_ is the peak value of the rate constant for CB attachment; g_1_ and g_2_ are the peak values of the rate constants for CB detachment; w is the maximum mechanical work of a unitary CB (w/e = 0.75); e is the free energy required to split one ATP molecule. The standard free energy ΔG°’_ATP_ was roughly—60 kJ/mole, and the e value used was 10^−19^ J [24].

N is the cycling CB number per mm^2^ at peak isometric tension. The molecular step size h is the translocation distance of the actin filament produced by the swing of the myosin head. The parameter l is the distance between two successive actin sites with which any myosin site could combine. In accordance with A. Huxley conditions (l >> h), the values of h and l were h = 10 nm and l = 28.6 nm, respectively [25,26,27,28]. Calculations of f_1_, g_1_, and g_2_ were made using the following equations:

G = f_1_/g_1_ (4)

g_1_ = 2wb/ehG (5)

g_2_ = 2Vmax/h (6)

kcat = (h/2l) × [(f_1_g_1_)(f_1_g_1_)] (7)

Po = (w/l) × [(f_1_)/(f_1_ + g_1_)] (8)

where **N**, the number of active CBs/mm^2^, is the ratio of the peak isometric tension and the unitary CB force (po). Myosin content (MC) was calculated from the CB number per g of tissue (nM/g) and the Avogadro number. The myosin ATPase activity was the product of the catalytic constant (kcat) and MC. The rate of mechanical work (W_M_) was equal to P_Hux_ × V. The efficiency of the contractile tissue was the ratio of W_M_ and E. Peak efficiency (Effmax) was the maximum value of efficiency (Figure 1B,C).

#### 2.4.3. Relaxation

We investigated two basic processes involved in the contractile mechanism, i.e., the actin–NM CB cycle inhibited by the 2,3-butanedione monoxime (BDM) and the activation of the NO-cGMP pathway with isosorbide dinitrate (ISDN).

#### 2.4.4. Statistical Analysis

Data were expressed as means ± standard deviations.

## 3. Results

### 3.1. Immuno-Histochemistry 

The immuno-histochemical analysis revealed expressions of SMA, NM-IIA, NM-IIB, and β-catenin (Table 1). In all tumor cells, membranous staining of β-catenin appeared. In the peritumoral stroma, β-catenin was also seen, but less clearly. Moreover, NM-IIA was detected in the peritumoral stroma in 95.2% of the samples (20/21 cases) and in tumor cells in 90.4% of the tumors (19/21 cases). As for NM-IIB, it was detected in 85.7% of the peritumoral stroma (18/21 cases) and in 90.4% of the tumor cells (19/21 cases). Alpha smooth muscle actin (SMA) was present in all peritumoral stroma cells but never in the tumor cells (Table 1).

**Figure 2:**
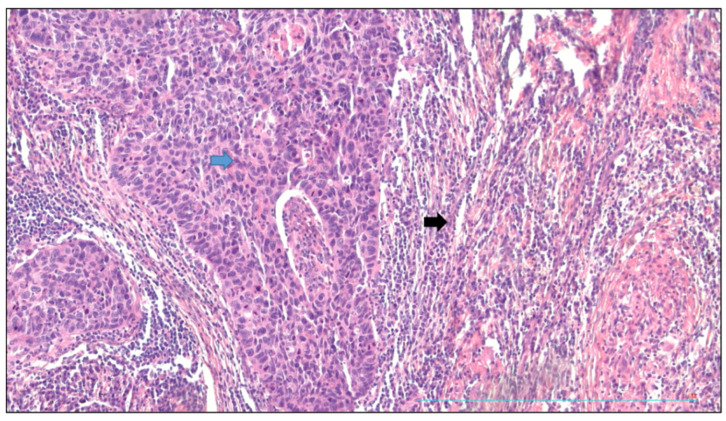
Squamous cell carcinoma of lung and peritumoral stroma. Magnification ×20, HES staining. The tumor proliferation (blue arrow) was composed of dense masses of cohesive squamous cells of tumor origin. It was surrounded by a peritumoral reactive stroma (black arrow) consisting of a fibrous background and a dense lympho-plasmacytic infiltrate.

Figure 2 shows the squamous cell carcinoma of the lung with tumor proliferation surrounded by a peritumoral reactive stroma; the sign − signified the absence; the sign + signified the presence.

**Figure 3:**
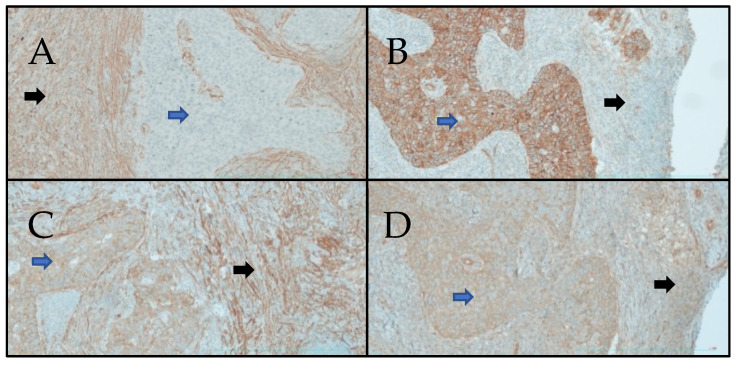
Expression of SMA, β-catenin, NM-IIB, and NM-IIA. Magnification ×20. (**A**) Staining of the tumor stroma by SMA (black arrow). There was no significant staining of tumor cells (blue arrow). Smooth muscle actin (SMA) marked the myofibroblasts. (**B**) β-catenin: tumor cells exhibited intense membranous staining of β-catenin (blue arrow). The stroma showed a weak staining (black arrow), compared with that of tumor cells. (**C**) NM-IIB: Membranous staining was observed in tumor cells (blue arrow) and peritumoral stroma (black arrow). (**D**) NM-IIA: Membranous staining was observed in tumor cells (blue arrow) and peritumoral stroma (black arrow).

Figure 3 represents the expression of SMA, β-catenin, NM-IIA, and NM-IIB in immuno-histochemistry.

### 3.2. Mechanics

#### 3.2.1. Contraction

**Figure 4:**
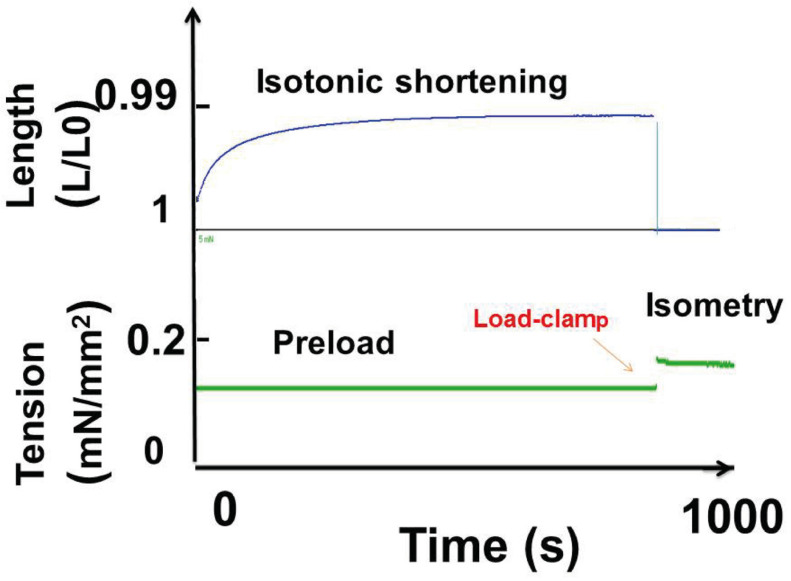
Parameters of contraction under tetanic stimulation: shortening length (blue curve) rapidly reached a maximum. Then, an isometric load clamp was imposed. The shortening length returned rapidly to the resting length and the tension curve (green) developed the maximum isometric tension.

Mechanical results were calculated in lung cancer cells under tetanic stimulation. Mechanical parameters are described in Figure 4. The shortening length reached asymptotical Lmin/Lo. An isometric load clamp made it possible to determine the isometric total tension.

Table 2 shows the main contractile parameters of the experimental samples and molecular properties of the actin–NMIIA and IIB myosins (left column). Tension/mm^2^ was very low due to the particularly weak concentration of NM myosin crossbridges. Likewise, kcat was low because of the weak values of the CB attachment and detachment constants. However, the CB individual force remained normal.

#### 3.2.2. The Frank–Starling Mechanism and Hyperbolic Hill Relationship

**Figure 5:**
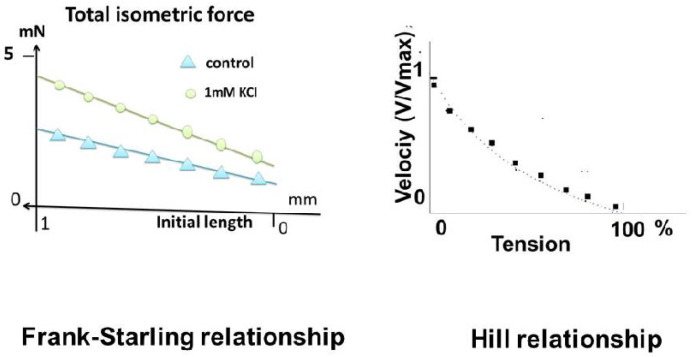
The Frank–Starling mechanism and the Hill hyperbolic relationship. (**Left**): The Frank–Starling curve represents the isometric tension as a function of initial length. Triangle: control values; circle: after inotropic effect (1 mM KCl). After the inotropic effect, the Frank–Starling curve was above the control curve and with a steeper slope. (**Right**): the Hill relationship. The tension–velocity (T–V) relationship describes a hyperbola.

The Frank–Starling mechanism was observed in all samples of lung cancers. It was characterized by an increase in the active isometric tension when the initial length increased. This means a certain degree of ultra-organization of the non-muscle myosins and actin filaments at the intracellular level (Figure 5, left). After a positive inotropic effect induced by KCl, the linear tension–length curve was shifted above the control curve with an increase in the slope and the origin ordinate.

#### 3.2.3. Tridimensional Tension–Velocity–Shortening-Length Relationship

**Figure 6:**
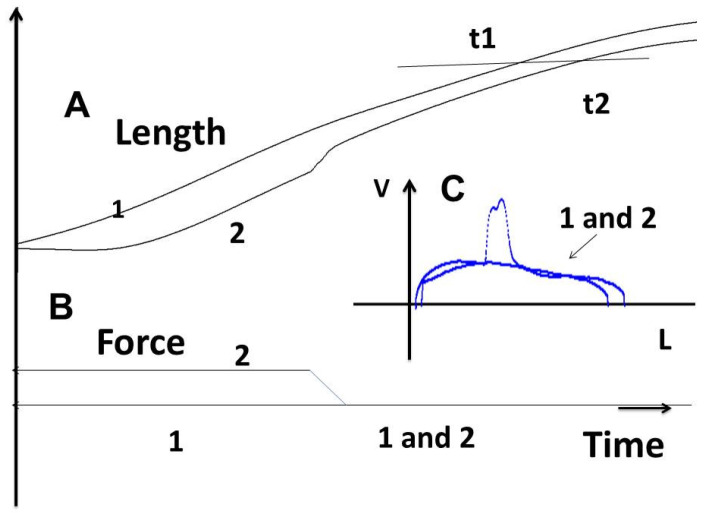
The tridimensional length–velocity–tension relationship. Length (**A**) and tension (**B**) are represented as a function of time; in addition, the phase-plane velocity–length (V–L) (**C**) was also drawn. Two curves were imposed with different loading conditions: curve 1 with a constant load (preload), and curve 2 with an afterload higher than the preload of curve 1 and clamped to the preload, i.e., at the same load level as that of curve 1. After a brief overshoot, the trajectories of curves 1 and 2 were superposed on the V–L phase-plane diagram (blue curve), but occurred at two different times on the length curve according to time (t1 and t2). On the V–L phase-plane (**C**) and after an overshoot, curve 2 followed the same trace as curve 1, before it dissociated from curve 1.

The tridimensional tension–velocity–length relationship is a fundamental property of muscle tissues. This property says that, in a part of the contraction (between the maximum velocity and the peak shortening length), the shortening velocity is a unique function of the shortening length, independently of time. Thus, a given degree of shortening length is always obtained with the same shortening velocity (Figure 6: t1 and t2). This is why it is referred to as “independent of time”. The tridimensional time-independent surface obtained with different afterloads defined the level of contractility of the mechanical sample. A new level of contractility, higher than that of controls, was obtained under 1 mM KCl.

#### 3.2.4. Relaxation

**Figure 7:**
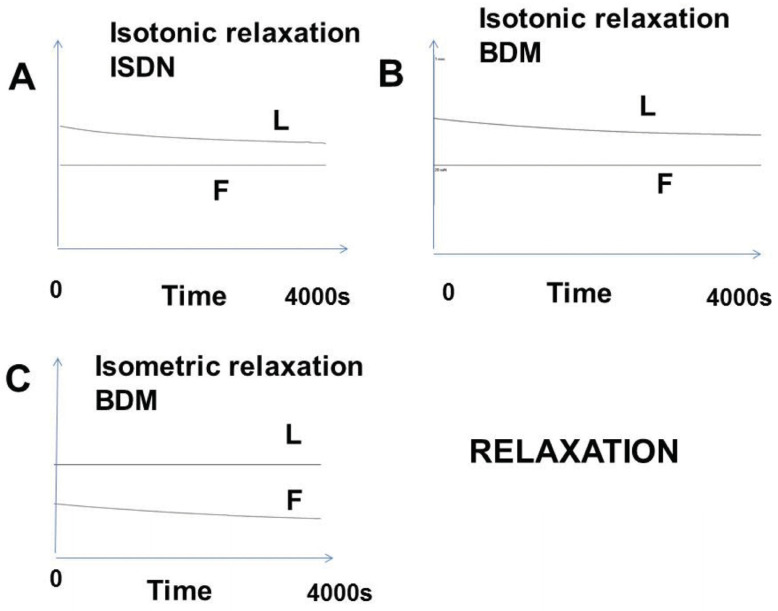
Relaxation of lung samples. L: Length; F: Force. (**A**) Isotonic relaxation obtained with ISDN; (**B**) isotonic relaxation obtained with BDM; (**C**) isometric relaxation obtained with BDM.

Relaxation was induced by activating the NO-cGMP pathway (ISDN) or by pharmacological agents inhibiting CB myosin molecular motors (BDM). As shown in Figure 7, the isotonic and isometric relaxations occurred very slowly (example: 4000 s).

## 4. Discussion

Up until now, when talking about contractile tissues, we included both sarcomeric skeletal and cardiac striated muscles and non-sarcomeric smooth muscles. Recently, the concept of non-muscle contractile tissues has emerged with tissues that are clearly not muscles but which exhibit contractile properties that share strong analogies with those of muscles themselves [29].

In the present study, the pathological lung parenchyma appeared to behave like a contractile tissue. It presented all the fundamental properties of muscles, except for the extremely slow kinetics. Thus, the following properties were found: (1) it was electrically stimulated by an electrical current or chemically with KCl; (2) it obeyed the Frank–Starling law, which states that the isometric tension increased with the initial length of the experimental specimen; (3) it obeyed Hill’s hyperbolic tension–velocity relationship; (4) for a given level of afterload, the instantaneous velocity of contraction was a unique function of the instantaneous length and this occurred independently of time. This property defines the level of contractility on the part of the three-dimensional time-independent force–velocity–length relationship. (5) It relaxed by decreasing the intracellular calcium or inhibiting contractile proteins. This is an effect due to the involvement of NMIIA and B (the SMA being the same as in the muscle systems). This allows a new classification of contractile systems: on the one hand, muscle with systems functioning with muscle myosin and, on the other hand, with non-muscle systems functioning with ultraslow NMIIA and B. These properties were found in all striated and smooth muscles but with much faster kinetics.

Table 2 shows the contractile parameters of the lung parenchyma cancers compared with those of two non-muscle preparations (normal placenta and bone marrow mesenchymal stem cells after differentiation by transforming growth factor-β (TGF-β)) and with a sarcomeric muscle (heart). Maximum shortening velocity, isometric tension, myosin content, kcat, and CB attachment and detachment rate constants were of the same order of magnitude in non-muscle preparations and in lung cancer. However, they were incomparably lower than the respective values observed in sarcomeric muscle (heart). On the other hand, the unitary CB force and maxEfficiency were of the same order of magnitude in non-muscles and muscle preparations.

The contractile profile observed here cannot be that of a smooth or striated muscle. Thus, in tracheo-broncho-pulmonary muscle tissues, the time for a contraction–relaxation cycle is rapid (order of magnitude: some seconds for a smooth muscle and less than one second for a striated muscle). In contrast, in lung non-muscle contractile cells, it was dramatically longer (order of magnitude: several thousands of seconds—Figure 4 and Figure 7). The duration of the contraction–relaxation cycle in non-muscle preparations (normal placenta and bone marrow mesenchymal stem cells) after differentiation by TGF-β is of the same order of magnitude. All these non-muscle preparations work with the same molecular motor, namely the non-muscle myosins, NMII A and B, that are coupled with the SMA [1]. NMII binds with actin through the head of the heavy chain where the ATPase site is located. NMII molecules assemble into bipolar filaments, which allows the sliding of the NM myosin molecules along the actin filaments (Figure 1C).

The main characteristic of NMII is its extremely slow kinetics [6]. Compared with striated or smooth muscles, the values for the CB detachment constant, the CB attachment constant, the catalytic constant, and the NMII-ATPase activity are dramatically low (Table 2). However, the NMII–CB unitary force is of the same order of magnitude when compared with MII.

The canonical WNT/β-catenin is often reported in diseases such as in human cancers [10]. It intervenes in multiple processes, namely in the immune cycle [14], the cell division cycle [11,12,13], and the immune cycle [14]. This was reinforced by the significant expression of the β-catenin in tumor cells (Figure 3B). With the significant expression of SMA (Figure 3A), this suggested the presence of myofibroblasts and the synthesis of fibrotic processes [16,17]. In the stroma, the myofibroblast membrane has the TGFβR1-2 receptor, which, when activated by TGFβ1, leads to the synthesis of β-catenin. The myofibroblast is the basic cell of non-muscle contractile tissues that was discovered by Gabbiani [30,31,32] during research on the presence of modified fibroblasts in the wound granulation tissue of healing skin. The contractile process is a retractile phenomenon associated during fibrosis with the synthesis of collagen in the extracellular matrix. This leads to irreversible fibrosis and apoptosis of myofibroblasts. As in wound healing or fibrotic processes, myofibroblasts generate a phenomenon of contraction–retraction with no relaxation [30,31,32]. From a therapeutic point of view, in cancers, the inhibition of the canonical WNT/β-catenin pathway should have a beneficial effect, especially when inflammatory processes are major.

## 5. Conclusions

In cancers, lung tissues acquire special contractile properties. These are similar to the contractile properties of muscles, except for their kinetics, which are ultraslow. We observed the Frank–Starling phenomenon, the hyperbolic tension–velocity relationship, the tridimensional time-independent force–velocity–length relationship, and the relaxation mechanism, all mechanisms described in muscles. Contractile properties were due to non-muscle myosin type IIA and IIB as shown in tumor stroma and in tumor cells. They were the molecular motors whose kinetic properties were particularly slow. The activation of the β-catenin canonical WNT pathway and synthesis of non-muscle myosin and SMA were associated as in several cancers and fibrosis processes.

## Figures and Tables

**Table 1:** Expression of SMA, NM-IIA, NM-IIB, and β-catenin in immuno-histochemistry of the histological type of tumors.

		NM-IIA		NM-IIB		SMA		β-Catenin	
n°	Histological Type	Tumor	Stroma	Tumor	Stroma	Tumor	Stroma	Tumor	Stroma
1	Adenocarcinoma	+	+	+	+	-	+	+	+
2	Adenocarcinoma	+	+	+	+	-	+	+	+
3	Adenocarcinoma	+	+	+	+	-	+	+	+
4	Squamous cell carcinoma	-	+	+	+	-	+	+	+
5	Adenocarcinoma	+	+	-	+	-	+	+	+
6	Adenocarcinoma	+	+	+	+	-	+	+	+
7	Adenocarcinoma	+	+	+	+	-	+	+	+
8	Carcinoid carcinoma	+	+	+	+	-	+	+	+
9	Adenocarcinoma	+	-	+	-	-	+	+	+
10	Adenocarcinoma	+	+	+	+	-	+	+	+
11	Adenocarcinoma	+	+	+	+	-	+	+	+
12	Squamous cell carcinoma	+	+	+	+	-	+	+	+
13	Prostatic adenocarcinoma	-	+	+	-	-	+	+	+
14	Squamous cell carcinoma	+	+	+	+	-	+	+	+
15	Adenocarcinoma	+	+	+	+	-	+	+	+
16	Adenocarcinoma	+	+	+	+	-	+	+	+
17	Squamous cell carcinoma	+	+	+	+	-	+	+	+
18	Adenocarcinoma	+	+	+	+	-	+	+	+
19	Adenocarcinoma	+	+	+	+	-	+	+	+
20	Squamous cell carcinoma	+	+	+	-	-	+	+	+
21	Adenocarcinoma	+	+	-	+	-	+	+	+

**Table 2:** Contractile parameters and molecular properties of NMII.

	Lung Cancer	Placenta	MSC	Heart
Vmax	0.003	0.002	0.002	3.7
Tension	0.2	1.5	0.3	42
kcat	0.004	0.003	0.004	20.6
CB force	2.0	2.0	2.1	1.6
max.Eff (%)	36	36	38	28
MC	0.01	0.14	0.07	12.5
G	4.5	3.7	4.1	1.6
f1	0.09	0.08	0.08	308
g1	0.04	0.03	0.03	194
g2	0.52	0.34	0.35	731

Parameters of contraction and myosin crossbridge characteristics. Vmax: Lmax/s; Tension: mN/mm^2^; kcat: s^−1^; CB force: pN; max.Eff (%): maximum efficiency in %; MC: myosin content; nM/g: G curvature; f1: rate constant for attachment in s^−1^; g1: rate constant for detachment in s^−1^; g2: rate constant for detachment in s^−1^. Blue is for lung in the present study; green is for ultra-slow non-muscle preparations in previous studies; red is for the cardiac muscle in a previous study.

## Data Availability

The data that support the findings of this study are available on request from the corresponding author. The data are not publicly available due to privacy or ethical restrictions.
